# Agreement Between Mothers and Fieldworkers While Assessing Child Development Using Ages and Stages Questionnaires, Third Edition in Nepal

**DOI:** 10.3389/fpsyg.2020.579412

**Published:** 2020-11-12

**Authors:** Merina Shrestha, Catherine Schwinger, Mari Hysing, Ram Krishna Chandyo, Manjeswori Ulak, Suman Ranjitkar, Ingrid Kvestad, Shakun Sharma, Laxman Shrestha, Tor A. Strand

**Affiliations:** ^1^Tribhuvan University Teaching Hospital, Kathmandu, Nepal; ^2^Centre for International Health Centre for Intervention Science in Maternal and Child Health, Faculty of Medicine, University of Bergen, Bergen, Norway; ^3^Department of Psychosocial Science, Faculty of Psychology, University of Bergen, Bergen, Norway; ^4^Kathmandu Medical College Teaching Hospital, Kathmandu, Nepal; ^5^Department of Global Public Health and Primary Care Centre for Intervention Science in Maternal and Child Health, Faculty of Medicine, University of Bergen, Bergen, Norway; ^6^Regional Centre for Child and Youth Mental Health and Child Welfare (RKBU), Bergen, Norway; ^7^Department of Research, Innlandet Hospital Trust, Lillehammer, Norway

**Keywords:** ASQ, home procedure, mail out, assessment, low income and middle income countries

## Abstract

**Background:** The Ages and Stages Questionnaires, Third Edition (ASQ-3) is becoming a widely used developmental assessment tool. The ASQ-3 can be completed by the caregivers (referred to as “mail out”), or by trained personnel under direct observation of the children (referred to as “home procedure”).

**Aim:** The study was carried out to compare results obtained by the ASQ mail out with those of the ASQ home procedure in a community setting of Bhaktapur, Nepal.

**Methods:** Trained fieldworkers (FWs) performed developmental assessment of 134 children aged 9 months in their homes using the ASQ home procedure. A few days before these assessments, mothers were asked to fill in the same ASQ-3 questionnaire. The concordance correlation coefficient (CCC) was calculated to measure their agreement.

**Result:** The agreement between the ASQ mail out and home procedure was fair for the total score (CCC = 0.54). For the sub-scales, the agreement was good for the gross motor (CCC = 0.65), for the remaining subscales agreement was poor (CCC < 0.4).

**Conclusion:** In resource limited setting like Nepal, the ASQ mail out represents an easy method to assess child development by caretakers at home; however, with the poor agreement between different methods of assessments, we cannot conclude that a single method is superior or most optimal and this question should be investigated further. When either of the method home procedure or mail out is opted, the results should be interpreted with cautions.

## Introduction

In recent years, the Ages and Stages Questionnaire (ASQ) has been used globally as a developmental screening tool (Singha et al., 2017). Jane Squires and Diane Bricker from the University of Oregon, United States, developed it in 1980 to screen early development among babies admitted to intensive care units. The first edition of ASQ was published in 1995 and is originally a parent-completed developmental screening questionnaire (Squires et al., 1998). After several revisions, the ASQ third edition (ASQ-3) was published in 2009. The ASQ-3 is suggested to be a cost-effective and easy-to-use tool to screen for developmental delay among children between 2 and 66 months (Singha et al., 2017; Small et al., 2019). The internal consistency has been acceptable (Squires and Bricker, 2009); however, more recent, translated, and adapted versions have had lower internal reliability, especially for the problem-solving and personal-social domains (Velikonja et al., 2017). The ASQ-3 has been translated into at least 23 different languages and is being used in diverse cultural settings; however, the extent to which the user-friendliness is applicable to different cultures and populations is still unknown (Filgueiras et al., 2013; Small et al., 2019).

Though the questionnaire originally was developed and validated to track child development and completed by the parents (Elbers and Macnab, 2008), the guidelines in the ASQ-3 suggest that it can be completed by professionals in collaboration with parents and caregivers (Squires and Bricker, 2009). However, a variety of different assessment methods were adopted while using ASQ-3 in low and middle income countries (LMICs). A recent review of 53 articles on the use of ASQ in LMICs, 51% used parent completion, independently or by interview, and a large proportion of those without parent completion used a trained assessor with direct observation (Small et al., 2019). The direct observation procedure was more common in the countries classified with the poorest economies. As an example, a study from North India shows that using a procedure with direct observations by trained assessors is a feasible approach to assess developmental status in young children (Kvestad et al., 2013). The inter-rater agreement between the assessors both during standardization and the quality control procedures during the study was high, although the Cronbach alpha coefficients varied by subtest, these showed mostly acceptable internal consistency. Moreover, the ASQ-3 scores collected by this procedure showed to be associated with other factors known to be related to early child development (Kvestad et al., 2015), which provide support for its validity.

In the current research, in what we have called the “*home procedure*,” the assessors observed the child’s activity and only when observation was not possible, the parents’ report was opted for to score the items. The *ASQ home procedure* has also been described in other studies from LMICs (Kvestad et al., 2013; van Heerden et al., 2017; Thorne-Lyman et al., 2019), arguing that it could serve as an alternative when caregivers may not be able to adequately complete the forms such as in uneducated populations. In our previous study in Bhaktapur, we adopted the ASQ *home procedure* performed by trained fieldworker (FW) using standardized materials. This study showed that the mean ASQ-3 score of the Nepalese children was consistently lower than the American norms, and that the scores were able to discriminate between children at risk of poor development (e.g., prematurity, low birth weight, and stunting) (Shrestha et al., 2019a). However, this procedure is more time and resource consuming than the parental report. An important question is how the *home procedure* relates to another assessment method we adopted, where caregivers themselves fill in the questionnaires, hereafter referred to as the “*mail out*” method. In the *mail out* method, parents were requested to read and understand the questions and interact and play with children with appropriate available toys. After that, they were requested to observe children’s behaviors in their home environment and then score the questionnaire. If this method would yield similar results to the *home procedure*, this could save important resources in an already resource poor setting.

Thus, the primary objective of the current study was to compare the results obtained by the ASQ *home procedure* with those of the ASQ *mail out* in a community setting of Bhaktapur, Nepal. We also aimed to measure the internal consistency of the questionnaire when the *home procedure* and *mail out* methods for assessment were adopted. Moreover, we also compared the mean score of the study children to the U.S. norm. As questionnaire-based developmental assessment tools are rarely used in community settings in Nepal, we also intended to explore the mother’s feedback on the ASQ-3 questionnaire. To address our objectives, we used data from a randomized-controlled trial on the effects of vitamin B12 supplementation on growth and neurodevelopment. In this trial, the *home procedure* was adopted for all children, and the *mail-out* method was done in a subset of children for this study purpose only.

## Materials and Methods

### Study Sample

We enrolled mothers who participated in a double-blinded randomized controlled trial (RCT) entitled “Supplementation of vitamin B_12_ in pregnancy and postpartum on growth and neurodevelopment in early childhood: A Randomized, Placebo Controlled Trial” registered under the number NCT03071666 at clinicaltrials.gov. The study was set in the city of Bhaktapur, 15 km east of the capital city Kathmandu, Nepal. The city and the surrounding communities are spread over 119 km^2^ with population of 340,066 where the majority belongs to the Newar ethnic group. The primary source of income for most of the families is agriculture. Other sources of income are small-scale self-businesses, daily wage earning, and other labor. Social indicators include ownership of land and houses. The traditional way of living in joint families is gradually shifting to a more modern way of living in nuclear families. Along with the increment in literacy rate in the country, Bhaktapur currently has a female literacy rate of almost 70%.

For the RCT, women aged 20–40 years, with an early pregnancy of less than 15 weeks of gestation and who planned to stay in the study site for at least 2 years, were included. Women with severe anemia (hemoglobin concentration < 7g/dl), chronic diseases such as tuberculosis, diabetes, hypo or hyperthyroidism, recurrent spontaneous abortions, and women with malnutrition (BMI < 18.5 and > 30) and women suffering from pernicious anemia requiring vitamin B12 supplementation and who already were on multivitamin supplementations containing vitamin B12 were excluded.

All the enrolled women received calcium, iron, and folic acid supplementation as recommended by the local gynecologists. They were randomized to receive either daily supplements with 50 μg of vitamin B12 or placebo from the time of enrollment until 6 months postpartum. The children were followed till 24 months to assess their neurodevelopmental outcome. For our study, we randomly selected mother-infant-dyads when the child had reached 9 months of age.

### Ages and Stages Questionnaires Third Edition (ASQ-3)

The ASQ-3 consists of 30 questions; six in each of the five subscales: communication, gross and fine motor, problem solving, and personal social skills. There are 21 versions of the ASQ-3 questionnaire, each adapted for a specific age range. For the current study, we used the questionnaire for 9 months. This questionnaire is recommended to be used for age range 9 months and 0 days to 9 months 30 days. Each of the domains of development is scored “Yes,” “Sometimes,” and “Not yet.”

### Translation and Cultural Adaptions

Before using the questionnaire, it was translated and adapted following standard official recommendations (Wild et al., 2005). The first author who has experience in the field of early child development did the English to Nepali translation. A Nepalese professor in English literature did the back translation to English. A Norwegian child psychologist then independently verified the back-translated English version. After several discussions in the team of psychologists and FWs, one adaption was made to the questionnaire (i.e., problem solving, question 4) where “Pat-a-cake” was change to “Taali Taali,” which is a more cultural appropriate play for Nepalese children.

#### ASQ *Home Procedure*

In the ASQ *home procedure*, trained FWs directly observed children during the assessment. For the assessment, locally available toys and materials were used. For holding and banging, inch-sized wooden cubes, and for pincer grasp, cheerios were used. When the performance could not be directly observed while filling out the questionnaires, the mothers were asked about their perception on the child’s abilities, and scores were based on the reports from the mother/caretaker.

#### ASQ *Mail Out*

In the ASQ *mail out*, mothers completed the ASQ-3 questionnaire. Completing the questionnaire for the mothers is supposed to take approximately 15–20 min (Squires and Bricker, 2009). In our study, we provided a hard copy of the questionnaire to the mothers and requested them to fill it themselves with help from other family members when necessary, but without help from the FW. The questionnaires in the *home procedure* and the *mail out* were identical.

### Data Collection

All the demographic information on the mother was obtained at the time of enrollment. Information on each child was obtained from the birth record.

The ASQ *home procedure* was performed on children at 9 months of age by the trained FWs in their homes. If the procedure could not be performed on the scheduled date, it was done within a timeframe of 5 days. FWs who were not involved in ASQ assessment visited the child at home 3–5 days prior to the scheduled ASQ *home procedure*. During the visit, FWs briefed about the ASQ *mail out* and requested the mothers to fill in the ASQ-3 questionnaire as completely and thoroughly as possible. Before filling the forms, parents were requested to observe the child’s activity, and even try out items with the child if necessary. Mothers were also requested to fill in a feedback questionnaire that indicated the time taken to fill the form. A four-point Likert-type scale was used to assess the understandability of the ASQ-3 questionnaire among the mothers.

The completed questionnaires were collected before the ASQ *home procedure*, and the scores were blinded to the FWs performing ASQ *home procedure*.

### Training and Standardization

For the ASQ-3 assessment, three FWs were recruited. These FWs already received training on applying the ASQ-3 in children of similar age. Before starting the assessment, we had hands-on refresher training. After that, standardization exercises were performed in 20 children. For the standardization, the first author served as the gold standard. During the standardization procedure, one of the FWs performed the assessment, whereas the other two FWs and the person who served as the gold standard observed and scored independently. The intra-class correlation coefficients (ICCs) were calculated to compare the interrater agreement between the FWs’ assessments with the gold standard during standardization procedure. The agreement between the gold standard and the FWs was found to be above 0.9 for the gross motor, communication, and problem-solving subscales and above 0.75 for the personal social and fine motor subscale indicating excellent and good interrater agreement respectively (Table 1).

**TABLE 1:** Inter-rater agreement between gold standard and fieldworkers (FWs) during standardization exercise for the ASQ “*home procedure*.”

Subscales of ASQ-3	Intra-class correlation coefficient
Communication	0.96
Gross Motor	0.92
Fine Motor	0.75
Problem Solving	0.96
Personal Social	0.78

*Note: Interrater agreement measures: poor = less than 0.4; fair = between 0.40 and 0.59; good = 0.60 and 0.74; excellent = 0.75 and 1.00.*

### Statistical Analysis

Each item of the subscales was coded according to the ASQ-3 manual: yes = 10, sometimes = 5, and not yet = 0. For missing items, the mean score of the individual on the sub-scale was imputed as recommended in the ASQ-3 manual (Squires and Bricker, 2009). The demographic data, the mother’s feedback on the questionnaire, and the ASQ-3 scores (both overall and for each subscale) are presented as means (SD) or percentages as appropriate. The Student’s *t*-test was applied to test the significance of the mean difference between scores in our study and U.S. norms. The observed items by the FW in the ASQ-3 that do not rely on the mother’s report are presented as total number and proportion of the total number of answers.

To compare the agreement between FWs’ and mothers’ scores, the ICC with one-way random effects models for single measurements was calculated. The interrater agreement was considered poor if it was equal or below 0.40; fair if between 0.40–0.59; good if 0.60–0.74; and excellent if 0.75–1.00 (Cicchetti, 1994). We also calculated Lin’s concordance correlation coefficient (CCC) (Lawrence and Lin, 1989) and graphically displayed the agreement in Bland and Altman plots (Bland and Altman, 1986). The internal consistency of the total score and subscales was measured with standardized Cronbach’s alpha (α). The internal consistency is considered high when α > 0.80; satisfactory when α = 0.60–0.80; and moderate when α = 0.40–0.59. The analysis was performed in Stata version 16 (Stata, College Station, TX, United States; Barnhart et al., 2007).

### Ethics

The primary study had obtained ethical approval from the Nepal Health Research Council in Nepal (NHRC, NHRC; 253/2016) and from the Regional Committee for Medical and Health Research Ethics in Norway (REK vest; reference number 2016/1620). For this study, written informed consent was obtained from the mothers. The latest version of the Declaration of Helsinki was implemented for obtaining the consent.

## Results

### Demographic Characteristics

We selected 134 mothers with 9-month-old babies sequentially from the primary study. The mean (SD) age of the mothers was 27 (3.8) years. Almost 95% of the mothers had an education level higher than grade 5. About one-third of the mothers (30%) were housewives. There was an almost equal distribution of boys and girls among the children, and their mean (SD) birth weight was 2976 (466) g. Twelve percent of the children were born preterm (Table 2).

**TABLE 2:** Demographic characteristics of the women included in a study comparing the scores from the ASQ “*home procedure*” and “*mail out*.”

Maternal characteristics	
Age of mother, years (SD)	27 (3.8)
**Maternal educational level, *n* (%)**	
Illiterate	1 (0.7)
1–5 years	6 (4.5)
6–10 years	52 (38.8)
11–12 (high school)	45 (33.6)
13–15 (bachelor level)	20 (14.9)
16–17 (master level)	10 (7.5)
**Occupation of the mother, *n* (%)**	
Housewife/agriculture	46 (34.3)
Daily wage earner	23 (17.2)
Business	33 (24.6)
Service	32 (23.9)
**Paternal characteristics**	
Age of father, years (SD)	30 (3.8)
**Paternal educational level, *n* (%)**	
Illiterate	0 (0.0)
1–5 years	6 (0.1)
6–10 year	63 (47.0)
11–12 (high school)	40 (29.9)
13–15 (bachelor level)	10 (0.2)
16–17 (master level)	15 (0.2)
**Occupation of the father, *n* (%)**	
Agriculture	6 (4.5)
Daily wage earner	22 (16.4)
Business	65 (48.5)
Government employee	2 (1.5)
Service	39 (29.1)
**Child characteristics**	
Male child, *n* (%)	65 (48.5)
Birth weight (g), mean (SD)	2976 (466)
**Gestational age at birth,** *n* (%)	
Preterm (≤37 weeks)	16 (12)
Term (>37 weeks)	118 (88)

During the *home procedure*, most of the items (about 85%) in the gross motor, fine motor, and problem-solving subscales were directly observed by the FWs. In the communication subscale, most of the items were scored based on interview with the mothers. In the personal social subscale, items like drinking from a cup, self-feeding, holding a toy, and letting go of a toy were mostly observed; the rest of the items were mostly scored by interview (Table 3).

**TABLE 3:** Number of items observed by FWs for scoring while performing ASQ “*home procedure*” (*n* = 134).

	*n* (%)
**Communication**	
Does your baby make monosyllable babble?	63 (47)
Does your baby imitate the sound back to you?	6(4)
Does your baby make two bi-syllable babbles?	39 (29)
Without showing, does your baby play one nursery game? E.g.,“bye-bye,” “clap your hands,” “come, come”)?	42(31)
Without showing gestures, does your baby follow one simple command, such as “go there,” “close your eyes,” or “sit down’?	16 (12)
Does your baby say three words, such as “Mama,” “Dada,” and “Baba”?	6 (4)
**Gross Motor**	
Does your baby support her/his weight to stand while holding hands just to balance?	113 (84)
Does your baby sit without support for several minutes?	113 (84)
Does your baby stand holding the furniture without leaning her chest against the furniture for support?	113 (84)
Does your baby bend down and pick up a toy and stand up while holding onto furniture?	113 (84)
Does your baby lower herself without falling while holding the furniture?	114 (85)
While holding the furniture with one hand, does your baby walk on the side of the furniture?	113 (84)
**Fine Motor**	
Does your baby pick up a small toy with only one hand?	115 (86)
Using her thumb and all of her/his fingers does your baby pick up a crumb or Cheerio?	114 (85)
Using tips of his thumb and fingers and with a space between the toy and the palm, does your baby pick up a small toy?	114 (85)
Does your baby pick up a piece of string with pincer grasp?	111 (83)
Does your baby pick up a crumb or Cheerio pincer grasp?	114 (85)
Does your baby take off the hand of the toy while putting it down?	115 (86)
**Problem Solving**	
Does your baby transfer objects from one hand to the other?	112 (84)
After picking up, does your baby hold a toy in each hand for about 1 min?	112 (84)
Does your baby bang a toy on the table with a toy in his hand?	115 (86)
Does your baby clap with a toy in each hand?	114 (85)
Does your baby try to get out or poke at a Cheerio that is inside a clear bottle?	112 (84)
Does your child find out a small toy hidden under a piece of cloth? (after watching you hide)	114 (85)
**Personal Social**	
Does your child put the foot in her/his mouth while lying on the back?	9 (6)
While you hold a cup, does your baby drink water from it?	111 (83)
Does your baby feed himself a biscuit?	107 (80)
When you ask to give the toy to you, does your child extend the hand even though he/she doesn’t let go of it?	104 (77)
While dressing the baby does he/she push the arm through a sleeve when arm reaches the hole of the sleeves?	14 (10)
When you ask to give the toy to you, does your child extend the hand and let go of it?	106 (79)

*Note: ASQ, Age and Stage Questionnaire.*

### ASQ Mean Score and Comparison With U.S. Norms

The total mean score obtained from the ASQ *home procedure* was 213.1 (31.7). When compared with U.S. norms, the Nepalese infants had significantly lower mean scores in all the subscales except in the communication subscale, where the average scores were significantly higher than the U.S. norms (Table 4).

**TABLE 4:** Mean (SD) scores in the Nepalese infants obtained with ASQ “*home procedure*” and ASQ “*mail out*” compared with the U.S. norm sample mean (SD).

	Mean (SD)	Difference (95% CI)†	
**Communication**			
*U.S. mean*	38.6 (12.9)	reference††	
*Home procedure*	44.1 (9.4)	5.5	3.3, 7.7*
*Mail out*	46.3 (9.4)	7.8	4.8, 10.7*
**Gross Motor**			
*U.S. mean*	46.7 (14.5)	Reference	
*Home procedure*	43.2 (15.3)	–3.5	−5.9, −1.1*
*Mail out*	40.6 (12.5)	–6.2	−9.4, −2.9*
**Fine Motor**			
*U.S. mean*	52.3 (10.5)	Reference	
*Home procedure*	47.4 (9.6)	–4.9	−0.6.7, −3.1*
*Mail out*	47.8 (9.9)	–4.5	−6.9, −2.1*
**Problem Solving**			
*U.S. mean*	49.5 (10.4)	Reference	
*Home procedure*	44.9 (31.1)	–4.6	−0.6.4, −2.8*
*Mail out*	45.4 (11.4)	–4.2	−0.6.8, −1.5*
**Personal Social**			
*U.S. mean*	42.5 (11.8)	Reference	
*Home procedure*	33.3 (11.9)	–9.1	−11.1, −7.1*
*Mail out*	33.4 (11.0)	–9.1	−11.8, −6.4*

^†^ CI, confidence interval.

*Significant difference *p* < 0.0001.

*^††^ Mean differences compared to U.S. mean.*

### Reliability Measures

**FIGURE 1:**
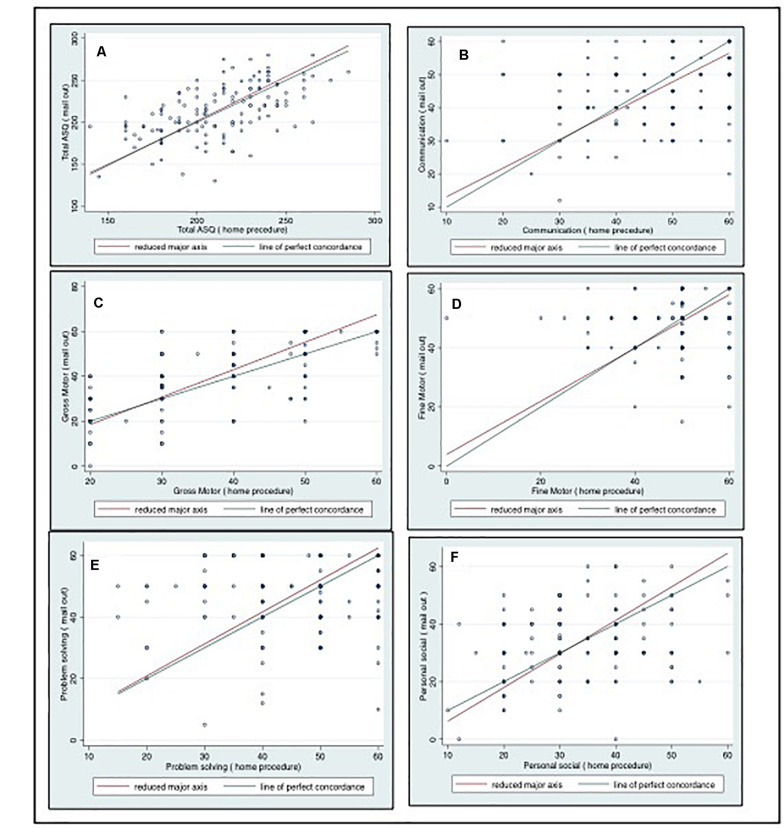
Bland and Altman’s limits-of-agreement procedure plots between ASQ-3 scores in 134 Nepalese children aged 9 months assessed by the fieldworkers (horizontal axis) and mothers (vertical axis) for **(A)** the total ASQ-3 score, **(B)** the communication subscale, **(C)** the gross motor subscale **(D)**, the fine motor subscale **(E)**, the problem solving subscale **(F)**, and the personal social subscale.

(i) Agreement between scores obtained from ASQ *home procedure* and ASQ *mail out*: The ICC/CCC between ASQ *home procedure* and ASQ *mail out* was fair for the total ASQ score (0.54), and good (0.65) for the gross motor subscale; in all the remaining subscales the agreement was poor (below 0.4) (Table 5).
  Figure 1 depicts the agreement between the scores of the mothers and the FW and the deviation of the observed data from the optimal line of concordance using the Bland and Altman’s limits-of-agreement procedure.
(ii) Internal Consistency of scores obtained from the ASQ *home procedure* and from the ASQ *mail out*: The standardized Cronbach alphas for the total score were 0.58 and 0.62 for *home procedure* and *mail out*, respectively, indicating a satisfactory internal consistency. For the subscales, the gross motor subscale had alphas for both methods were 0.6 indicating satisfactory internal consistency. The remaining subscales both for FW and mothers had alphas below 0.6 indicating poor internal consistency (Table 6).

**TABLE 5:** Participants’ mean score, intra-class correlation coefficients (ICC), and concordance correlation coefficient (CCC) between ASQ “*home procedure*” and ASQ “*mail out*.”

*N* = 134	FW (SD)	Mother (SD)	ICC (95% CI)	CCC (95% CI)
Total ASQ-3	213.1 (31.7)	213.4 (30.8)	0.54 (0.4, 0.6)	0.54 (0.4, 0.7)
Communication	44.1 (9.4)	46.3 (11.4)	0.28 (0.1, 0.4)	0.27 (0.1, 0.4)
Gross Motor	43.2 (15.3)	40.6 (12.5)	0.65 (0.5, 0.7)	0.66 (0.6, 0.8)
Fine motor	47.4 (9.6)	47.8 (9.9)	0.03 (0.0, −0.2)	0.04 (−0.1, 0.2)
Problem solving	44.9 (31.1)	45.4 (11.4)	0.05 (0.0, 0.2)	0.07 (−0.1, 0.2)
Personal Social	33.3 (11.9)	33.4 (11.0)	0.26 (0.1, 0.4)	0.27 (0.1, 0.4)

*ICC poor if < 0.40; fair if between 0.40–0.59; good if 0.60–0.74; and excellent if 0.75–1.00.*

**TABLE 6:** Standardized Cronbach’s alphas^a^ for the total ASQ-3 score and the subscale scores for each age range (*n* = 134).

	FW	Mother
Total ASQ-3 Score	0.58	0.62
Communication	0.27	0.40
Gross Motor	0.65	0.61
Fine Motor	0.37	0.49
Problem Solving	0.55	0.55
Personal Social	0.49	0.38

*^*a*^α > 0.80—high; α = 0.60–0.80—satisfactory; and α = 0.40–0.60—moderate internal consistency.*

### Mothers’ Feedback on the ASQ-3 Questionnaire

About 80% mothers responded that the questions alerted them about their child’s activity that they were not sure about, and that they gained new ideas about how to interact or play with their child. They also responded that the questions were easy to understand but they also mentioned that the communication and fine motor subscale questions were the most difficult among the subscales.

The mean time taken for the mothers to complete the questionnaire was 15 min (range: 2–90 min). About two-thirds of the mothers completed the questionnaire within 10 min.

## Discussion

In the current study, we sought to describe the agreement between the ASQ-3 scores recorded by caregivers *(mail out*) and FWs (*home procedure*) in Nepalese infants. Although the mean scores between mothers and FWs were similar, we found the agreement between them to be fair for the total ASQ score. In the individual subscales, the agreement was good for the gross motor subscale only, and poor for the rest of the subscales.

The good agreement in the gross motor subscale might be because mothers had better knowledge on gross motor development as compared to other domains of development, which was previously also shown in the same community (Shrestha et al., 2019b). Gross motor functions and abilities may also be easier to observe in this age group, hence, improving the agreement between mothers and FWs. Similar finding showed that parental report on gross motor milestone is trustworthy in young children (Bodnarchuk and Eaton, 2004). Other subscales like fine motor and problem solving showed poorer agreement with FWs’ scores. In the fine motor subscale, each item requires very meticulous observation, e.g., items such as “picking the toys with thumb and fingers” and “thumb and index finger” and “visible space between fingers and palms.” Therefore, there is always a chance of overlooking details in these items, in particular when one is not trained to score them. It is also seen that the internal consistencies between items were poor for some of the subscales like communication and fine motor. This was also reported in a study from Turkey, which showed lower internal consistency for the communication and problem solving subscale. The internal consistency for full scale improved with age with Cronbach alpha of 0.64 for 4 months to 0.92 for 60 months (Kapci et al., 2010).

To complete the ASQ questionnaire, caregiver/parents should be able to read and understand the questionnaire and it is generally assumed that mothers with education of minimum 4 years can easily score the ASQ-3. In our study, 95% of the mothers had this level of education. However, the quality and content of education might be different in different countries and context. The previously mentioned study in this population showed that mothers had limited knowledge on development in young infants, especially fine motor development, irrespective of their education level (Shrestha et al., 2019b). The limited knowledge and experience in filling out these types of questionnaires might have caused difficulties in understanding the nuances between the items resulting in the fair to poor agreement between FWs and mother’s score. Another reason for the poor agreement might be because of the relatively young age of the participating children. It has been shown that the ASQ-3 when compared with Bayley Scales of Infant and Toddler Development (BSID) III has lower sensitivity when applied to younger children (2–16 months) than in children above 18 months (Steenis et al., 2015). Also, other studies found that the validity of the ASQ improves with increasing age (Rubio-Codina et al., 2016).

In the communication subscale, mothers’ scores were on average higher than the FWs’ scores. These scores depend on their observation of the child over a long period in a familiar and non-alarming setting. In previous studies from the same study setting, we observed that in the presence of unfamiliar persons or in a test setting children vocalize less than usual (Ranjitkar et al., 2018; Ulak et al., 2020). In our study, most items in the communication subscale were scored by the FWs based on interview of the mothers. FWs were trained to produce the sound for the mothers for them to properly understand the item before providing the answer. We assume that when mothers completed the questionnaire themselves, it is possible that they might have found it hard to differentiate some of the items such as those concerning monosyllable and bi-syllable babbling. This might have resulted in higher scores in the communication subscale in the ASQ *mail out* compared to the ASQ *home procedure*.

When ASQ subscales scores of our Nepalese children obtained by the FW were compared with the U.S. normative sample, all subscale scores, except for the communication subscale, were lower compared with the U.S. normative sample in the present study. A similar finding was observed when children from the same community aged 6–12 months were compared in another study (Shrestha et al., 2019a). Normative scores for Nepalese children are not yet developed, and a direct comparison of the scores with U.S. norms is challenging. The low scores in the current study might be due to cultural aspects related to some of the items. For example, in the personal-social subscale, items such as “putting foot in the mouth” are considered as a dirty act and children are not allowed to do so. Similar cultural difference in the personal-social scale was noted from other LMICs as well (Gladstone et al., 2008).

The finding that scores on the communication subscale is higher in the Nepalese sample than the U.S. norm sample is striking, and in contrast to earlier findings from the same study settings where communication skills are lower in the Nepalese infants compared to U.S. norms (Ranjitkar et al., 2018; Shrestha et al., 2019a). The Georgian children were also found to have higher score than the U.S. sample in the communication subscale (Zirakashvili et al., 2018). In the U.S. norms, the mean scores for this subscale have a sudden drop compared to the mean scores for the age group above and below (Squires and Bricker, 2009). Hence, we cannot rule out that this finding is due to characteristics with the norm sample, and not that the Nepalese study children are performing comparably better. Also, in this subscale, differences in culture and language-related aspects could introduce biased mean scores.

Many mothers completed the questionnaire in 10 min. For the mothers who have never been exposed to these types of questions regarding child development, completing the questionnaire in such a short time might point to a less careful and thorough completion. Hence, it is possible that mothers might not have accurately reported on their children’s abilities. When items are unclear, there could be a tendency to simply agree to the question (Fernald et al., 2009). As 70% of the mothers work out of home, there might be lack of interactive parenting practices leading the mothers to misinterpret their children’s abilities. Another possibility might be that the mothers were scoring the items based on their past observations and memories, rather than based on real-time observations. Since the questionnaire’s responses were limited to “Yes,” “Sometimes,” and “Not Yet,” there is also a chance of under- or over-scoring. When children fail to perform, parents might overvalue their child’s developmental performance to avoid this perceived failure. The utilization of the ASQ-3 in itself can raise anxiety leading to distortion of the results or at times incomplete filling of the questionnaire (Kendall et al., 2019).

If mothers are trained and involved in developmental assessment, this could increase the interest of mothers in promoting the development of their children, and also their sensitivity to any lack or deficit in their child’s growth and developmental stage (Roshanfekr et al., 2017). Mothers, when involved in early stimulation interventions, could reach a better understanding of their child’s development and be able to implement the intervention strategies and become more compliant (Baker-Henningham and López Bóo, 2010).

### Limitations

First, the ASQ *mail out* was not compared with a clinical psychologist or a developmental pediatrician who are expert in the field. Though the FWs were trained, over the period of time, there is a chance of drift in the FWs scoring as seen previously (Shrestha et al., 2019a). Second, the assessments of the FW and the mothers were not completely independent as the mothers filled out the questionnaires before the FW’s assessment and in the *home procedure* some of the items were relied on interview of the mothers about the child’s ability. The number of reported items varies across the subscales and might thus have affected the agreement. Furthermore, there was no consistent pattern between the proportion of observed items and interrater agreement. Third, the study includes only young infants of 9 months, thus results cannot be generalized to other age groups.

## Conclusion

The current study was conducted to compare results obtained by the ASQ *home procedure* with those of the ASQ *mail out* in a community setting of Bhaktapur, Nepal. The agreement between the assessment methods as well as the internal consistency was poor for all sub-scales except the gross-motor sub-scale. With the poor agreement between different methods of assessments, we cannot conclude that a single method is superior or most optimal and this question should be investigated further. When either of the method *home procedure* or *mail out* is opted, the results should be interpreted with cautions.

## Data Availability Statement

The raw data supporting the conclusions of this article will be made available by the authors, without undue reservation.

## Ethics Statement

The main study has obtained ethical approval from the Nepal Health Research Council in Nepal (NHRC, NHRC; 253/2016) and from the Regional Committee for Medical and Health Research Ethics in Norway (REK vest; reference number 2016/1620). The patients/participants provided their written informed consent to participate in this study.

## Author Contributions

MS, MH, IK, RC, and TS designed the study. MU, RC, SR, SS, LS, and MS conducted the research and were responsible for the field implementation and data collection. MS, CS, and SR analyzed the data and interpreted the results. MS, CS, and TS had primary responsibility for the final content. All the authors read and approved the final manuscript.

## Conflict of Interest

The authors declare that the research was conducted in the absence of any commercial or financial relationships that could be construed as a potential conflict of interest.

## References

[B1] Baker-Henningham H. López Bóo F. (2010). “Early childhood stimulation interventions in developing countries: A comprehensive literature review,” in *IDB Publications (Working Papers) 2660* (Washington, DC: Inter-American Development Bank).

[B2] Bland J. M. Altman D. (1986). Statistical methods for assessing agreement between two methods of clinical measurement. *Lancet* 327 307–310. 10.1016/s0140-6736(86)90837-8 2868172

[B3] Bodnarchuk J. L. Eaton W. O. (2004). Can parent reports be trusted?: Validity of daily checklists of gross motor milestone attainment. *J. Appl. Devel. Psychol.* 25 481–490. 10.1016/j.appdev.2004.06.005

[B4] Cicchetti V. D. (1994). Guidelines, criteria, and rules of thumb for evaluating normed and standardized assessment instruments in psychology. *Psychol. Asses.* 6:284 10.1037/1040-3590.6.4.284

[B5] Elbers J. Macnab A. (2008). The Ages and Stages Questionnaires: feasibility of use as a screening tool for children in Canada. *Canad. J. Rural Med.* 13:493. 18208647

[B6] Fernald L. C. Kariger P. Engle P. Raikes A. (2009). *Examining early child development in low-income countries: a toolkit for the assessment of children in the first five years of life.* Washington, DC: World Bank.

[B7] Filgueiras A. Pires P. Maissonette S. Landeira-Fernandez J. (2013). Psychometric properties of the Brazilian-adapted version of the Ages and Stages Questionnaire in public child daycare centers. *Early Hum. Dev.* 89 561–576. 10.1016/j.earlhumdev.2013.02.005 23507472

[B8] Gladstone M. J. Lancaster G. A. Jones A. P. Maleta K. Mtitimila E. Ashorn P. (2008). Can Western developmental screening tools be modified for use in a rural Malawian setting? *Arch. Dis. Child* 93 23–29. 10.1136/adc.2006.095471 17379661

[B9] Kapci E. G. Kucuker S. Uslu R. I. (2010). How applicable are Ages and Stages Questionnaires for use with Turkish children? *Top. Early Child. Spec. Educat.* 30 176–188. 10.1177/0271121410373149

[B10] Kendall S. Nash A. Braun A. Bastug G. Rougeaux E. Bedford H. (2019). Acceptability and understanding of the Ages & Stages Questionnaires^®^, as part of the Healthy Child Programme 2−year health and development review in England: Parent and professional perspectives. *Child Care Health Devel.* 45 251–256. 30661256 10.1111/cch.12639 PMC6849765

[B11] Kvestad I. Taneja S. Hysing M. Kumar T. Bhandari N. Strand T. A. (2015). Diarrhea, stimulation and growth predict neurodevelopment in young North Indian children. *PLoS One* 10:e0121743. 10.1371/journal.pone.0121743 25826376 PMC4380317

[B12] Kvestad I. Taneja S. Kumar T. Bhandari N. Strand T. A. Hysing M. (2013). The assessment of developmental status using the Ages and Stages questionnaire-3 in nutritional research in north Indian young children. *Nutr. J.* 12:50. 10.1186/1475-2891-12-50 PMC3637815 23617745

[B13] Lawrence I. Lin K. (1989). A concordance correlation coefficient to evaluate reproducibility. *Biometrics* 45 255–268. 10.2307/2532051 2720055

[B14] Ranjitkar S. Kvestad I. Strand T. A. Ulak M. Shrestha M. Chandyo R. K. (2018). Acceptability and reliability of the bayley scales of infant and toddler development-III among children in Bhaktapur, Nepal. *Front. Psychol.* 9:1265. 10.3389/fpsyg.2018.01265 30087639 PMC6066572

[B15] Roshanfekr P. Gharibzadeh S. Mohammadinia L. Sajedi F. Habibi E. Malekafzali H. (2017). Involving mothers in child development assessment in a community-based participatory study using ages and stages questionnaires. *Int. J. Prevent. Med.* 8:102 10.4103/ijpvm.ijpvm_268_17 PMC5738788 29291044

[B16] Rubio-Codina M. Araujo M. C. Attanasio O. Muñoz P. Grantham-McGregor S. (2016). Concurrent validity and feasibility of short tests currently used to measure early childhood development in large scale studies. *PLoS One* 11:e0160962. 10.1371/journal.pone.0160962 27548634 PMC4993374

[B17] Shrestha M. Strand T. A. Ulak M. Chandyo R. K. Ranjitkar S. Hysing M. (2019a). The feasibility of the A ges and S tages Q uestionnaire for the assessment of child development in a community setting in Nepal. *Child Care Health Devel.* 45 394–402. 10.1111/cch.12654 30818415

[B18] Shrestha M. Ulak M. Strand T. A. Kvestad I. Hysing M. (2019b). How much do Nepalese mothers know about child development? *Early Child Devel. Care* 189 135–142. 10.1080/03004430.2017.1304391

[B19] Singha A. Yeh C. J. Blanchard S. B. (2017). Ages and Stages Questionnaire: a global screening scale. *Bol. Med. Hosp. Infant. Mex.* 74 5–12. 10.1016/j.bmhimx.2016.07.008 29364814

[B20] Small J. W. Hix−Small H. Vargas−Baron E. Marks K. P. (2019). Comparative use of the Ages and Stages Questionnaires in low−and middle−income countries. *Devel. Med. Child Neurol.* 61 431–443. 10.1111/dmcn.13938 29926467

[B21] Squires J. K. Potter L. Bricker D. D. Lamorey S. (1998). Parent-completed developmental questionnaires: Effectiveness with low and middle income parents. *Early Child. Res. Q.* 13 345–354. 10.1016/s0885-2006(99)80043-x

[B22] Squires J. Bricker D. (2009). *Ages & Stages QuestionnairesThird Edition (ASQ-3).* Baltimore: Brookes Publishing Company.

[B23] Steenis L. J. Verhoeven M. Hessen D. J. Van Baar A. L. (2015). Parental and professional assessment of early child development: the ASQ-3 and the Bayley-III-NL. *Early Hum. Devel.* 91 217–225. 10.1016/j.earlhumdev.2015.01.008 25703316

[B24] Thorne-Lyman A. L. Shrestha M. Fawzi W. W. Pasqualino M. Strand T. A. Kvestad I. (2019). Dietary Diversity and Child Development in the Far West of Nepal: A Cohort Study. *Nutrients* 11:1799. 10.3390/nu11081799 31382653 PMC6722734

[B25] Ulak M. Ranjitkar S. Shrestha M. Braarud H. C. Chandyo R. K. Shrestha L. (2020). The Feasibility of the Full and Modified Versions of the Alarm Distress Baby Scale (ADBB) and the Prevalence of Social Withdrawal in Infants in Nepal. *Front. Psychol.* 11:2025 10.3389/fpsyg.2020.02025 PMC7479187 32982842

[B26] van Heerden A. Hsiao C. Matafwali B. Louw J. Richter L. (2017). Support for the feasibility of the ages and stages questionnaire as a developmental screening tool: a cross-sectional study of South African and Zambian children aged 2-60 months. *BMC pediatr.* 17:55. 10.1186/s12887-017-0802-3 28209131 PMC5312428

[B27] Velikonja T. Edbrooke-Childs J. Calderon A. Sleed M. Brown A. Deighton J. (2017). The psychometric properties of the Ages & Stages Questionnaires for ages 2−2.5*: a systematic review*. *Child Care Health Devel.* 43 1–17. 10.1111/cch.12397 27554865

[B28] Wild D. Grove A. Martin M. Eremenco S. McElroy S. Verjee-Lorenz A. (2005). Principles of Good Practice for the Translation and Cultural Adaptation Process for Patient-Reported Outcomes (PRO) Measures: report of the ISPOR Task Force for Translation and Cultural Adaptation. *Val. Health* 8 94–104. 10.1111/j.1524-4733.2005.04054.x 15804318

[B29] Zirakashvili M. Gabunia M. Tatishvili N. Ediberidze T. Lomidze G. Chachava T. (2018). Cultural adaptation and psychometric validation of the ages and stages questionnaires for use in Georgia. *J. Child Fam. Stud.* 27 739–749. 10.1007/s10826-017-0917-z

